# Association between Paediatric Complementary and Alternative Medicine Use and Parental Health Literacy, Child Health, and Socio-Economic Variables: A Prospective Study

**DOI:** 10.3390/pediatric16020032

**Published:** 2024-05-08

**Authors:** Abida Denny, Andrew S. Day, Angharad Vernon-Roberts

**Affiliations:** 1 Otago Medical School, University of Otago, Dunedin 9016, New Zealand; 2 Department of Paediatrics, University of Otago, Christchurch 8011, New Zealand; andrew.day@otago.ac.nz

**Keywords:** children, complementary medicine, alternative medicine, health literacy, well-being

## Abstract

Complementary and Alternative Medicines (CAMs) constitute products and practices not considered allopathic medicine. CAM use is high in children, but little is known about factors that may influence parents using CAM with their child. This study aimed to determine the variables associated with CAM use in children with a prospective study among children and their parents attending a tertiary care hospital in New Zealand (NZ). Outcomes included current CAM use, parental opinions on CAM, parental health literacy and child well-being. This study was completed by 130 parents (85% female), and the mean child age was 6.7 years. CAM use was reported for 59 (45%) children, the most common being oral supplements and body manipulation. Children were more likely to use CAM if their parent had higher health literacy (*p* = 0.001), and if they had previously attended the emergency department within 12 months (*p* = 0.03). There was no association between child well-being and CAM use. Parental opinion of using CAM only if a doctor recommended it was associated with CAM use for their child (*p* = 0.01). Only 40% of parents disclosed their child’s CAM use to the medical team. This study highlights that parental health literacy influences the use of CAM for children in NZ, providing insight for translational research to improve CAM safety and disclosure rates in NZ.

## 1. Introduction

The term Complementary and Alternative Medicine (CAM) describes a diverse range of products and practices considered to be outside of conventional medical practice, which are used to support or replace allopathic treatments [1]. The reported rates of CAM use among the general paediatric population vary greatly between 11 and 87% [2,3]. CAM is more commonly used by children with acute and chronic health conditions [4,5,6,7]. Disclosure rates of a child’s CAM use to their treating medical team are often reported to be low, ranging from 0.7% to 42% [8,9]. The reasons for non-disclosure of such information are multifactorial, with origins in parental beliefs of efficacy, safety, and relevance, as well as clinician enquiry and support [10]. Non-disclosure of CAM use to a child’s clinical team can increase the risk of adverse outcomes such as contraindications and poor compliance with prescribed treatments [11,12,13]. Previous research has shown that ethnicity, taking prescription medication, overall child health, parental income and education, and family use of CAM may impact the use of CAM for a child [10,14,15].

An additional variable yet to be fully explored for its association with CAM use for children is parental health literacy. Health literacy is defined by experts as the extent to which people can obtain, process, and understand basic health information in order to make appropriate health decisions [16]. There is increasing evidence to suggest that poor parental health literacy can have negative effects on the health outcomes of children, especially regarding vaccinations, lifestyle, and medication administration [17,18,19,20,21,22]. The association between parental health literacy and paediatric CAM use is not widely studied. One study conducted in Turkey, for example, reported that a high proportion of parents who used honey for their children under one year old as a form of CAM exhibited poor health literacy [20].

In New Zealand (NZ), there are few resources available regarding the use of CAM for adults or children, despite between 56 and 70% of children using CAM [10,23]. CAM therapies are poorly regulated in New Zealand with limited legislation [24,25], although guidelines have recently been published for healthcare practitioners [26,27,28,29]. The capacity to carry out CAM research in NZ is limited by the availability of research supervision and support, resources such as time and funding, and the lack of a professional body for CAM practitioners [30]. Cultural barriers have been outlined as perceptions of negative bias towards CAM, and the holistic approach being misaligned with the reductionist approach to mainstream medicine [30]. This situation is evident despite widespread CAM use throughout the country [31], which is mirrored in the general population in Western countries [32,33] and developing countries [34], thereby highlighting the importance of identifying factors that may influence CAM use in children. The objective of the current study was to measure CAM use among children attending a tertiary care hospital in NZ alongside a number of child, family, and socio-economic variables. This study aimed to determine whether CAM use by children was associated with parental opinions and health literacy, socio-economic outcomes, healthcare utilization, and the physical, social, and mental health domains of a child’s health. 

## 2. Materials and Methods

### 2.1. Recruitment

Children and their parents were approached to participate via face-to-face recruitment throughout the inpatient and outpatient clinical areas of a tertiary care hospital: Christchurch Hospital, Christchurch, New Zealand. Data collection took place over five consecutive weeks during November to December 2023.

### 2.2. Study Population

Parents of children aged from birth to seventeen years were approached to participate. There were no exclusion criteria imposed.

### 2.3. Outcomes Measures

The following information was collected for analysis:

#### 2.3.1. Demographic Information

- Child age and sex;
- Parent age and sex;
- Parental education level;
- Family ethnicity;
- Residential address;
- Annual household income;
- Family structure.

#### 2.3.2. Child Health Information

- Prescription medications;
- Chronic health conditions;
- Reason for hospital attendance;
- Overall health in the last week (0–10 scale).

#### 2.3.3. Healthcare Visits

- Primary care physician visits within the previous 12 months;
- Emergency department visits within the previous 12 months;
- Outpatient visits within the previous 12 months.

#### 2.3.4. CAM Use

Parents were provided a sheet that displayed comprehensive examples of CAM types and categories, and then asked for information on the following variables.

- Child CAM use;
- If yes, type, frequency and duration;
- Reason for CAM use;
- CAM efficacy and side effects;
- Disclosure of CAM to medical team;
- Parental CAM use;
- Sibling CAM use.

#### 2.3.5. Parental Opinion Survey

Regardless of whether each participant’s child used CAM or not, all parents completed a survey about CAM use and acceptance. The survey comprised 16 statements answered using a five-item Likert scale ranging from strongly disagree to strongly agree. This survey was developed from assessment tools available in the literature and used in a previous research study [10]. 

#### 2.3.6. Health Literacy Assessment

All parents completed a validated health literacy assessment tool called the Newest Vital Sign [35,36], comprising a quiz that asks six questions about an ice cream nutrition label. Parents were given the label to view and then asked the six questions by the researcher. This was scored by giving one point for each correct answer to a maximum of six points, with levels of 0–1 indicating a high likelihood of limited literacy, scores 2–3 the possibility of limited literacy, and scores 4–6 adequate literacy [35]. 

#### 2.3.7. Child Well-Being

Child well-being was assessed using the Patient-Reported Outcomes Measurement Information System Parent Proxy Profile—25 (PROMIS-25) [37] that assesses parent-reported outcomes for their child within the domains of pain, fatigue, physical functioning, emotional distress, and social participation. As the PROMIS-25 parent proxy is only validated for children 5 years old and over, parents with children under this age were not asked to complete this measure. The version PROMIS^®^ Parent Proxy Profile v2.0—Profile-25 was used with the permission of the copyright holders.

#### 2.3.8. Socio-Economic Deprivation

Each participant’s residential address was collected and used to define deprivation deciles associated with the area the child resided using the New Zealand Indices of Multiple Deprivation [38]. This assigns deprivation deciles ranked from 1 (lowest deprivation score) to 10 (highest deprivation score) within the domains of overall deprivation, employment, income, crime, housing, health, education, and geographical access. 

### 2.4. Ethics and Consent

All children [aged > five years] provided their written assent for parents to provide information on them, and all parents provided written consent for participation. This study received ethical approval from the University of Otago Human Ethics Committee (Health) (H21/028) and Christchurch Hospital Locality Approval (RO21039).

### 2.5. Statistical Analysis 

Group comparisons between categorical and linear variables were performed by analysis of variance. A comparison of proportions between categorical variables was carried out using the Chi-squared (χ^2^) test of independence with results presented as χ^2^ value and the Phi effect size, with results closer to 1.0 indicating a stronger effect size. For the parental opinion survey, the five-point Likert scale responses were condensed to ‘Agree’, ‘Neutral’, and ‘Disagree’.

For the PROMIS-25 parent proxy survey, the total raw summed score was translated into a T-score for each participant for each domain using the PROMIS paediatric and parent proxy profile instrument scoring manual. The T-score rescaled the raw score into a standardized score with a mean of 50 and a standard deviation (SD) of 10. A higher PROMIS T-score represents more of the concept being measured, negatively worded domains (anxiety, depressive symptoms, fatigue, and pain interference) indicate worse outcomes, and positively worded concepts (mobility, peer relationships) indicate better outcomes. 

The deprivation decile data were presented as percentage difference (observed–expected) between cohort and known regional data from District Health Board (DHB) data [39], with differences between CAM users/non-users assessed using Chi-squared goodness of fit. Deprivation deciles for children using CAM were compared to those not using CAM by comparison of medians using Mann–Whitney U tests. 

Significance was considered at a level < 0.05. Analysis was performed using SPSS Statistics for Windows, Version 29.0 (IBM Corp., Armonk, NY, USA), and select graphs were created using Microsoft Excel™ Version 16, 365 Enterprise (Microsoft Corporation, United States).

### 2.6. Sample Size Calculation

Previous work has studied CAM use among children attending a tertiary care hospital in New Zealand, reporting a point prevalence of 56% from a population of 236 children [10]. An accurate sample size calculation was, therefore, available for adequate precision with 92 children required to be recruited (±8%, 95% CI).

## 3. Results

### 3.1. Patient Demographics

One hundred and thirty parents completed the survey, reporting data on 130 children (Table 1).

### 3.2. CAM Type, Duration, Frequency, and Side Effects 

**Figure 1:**
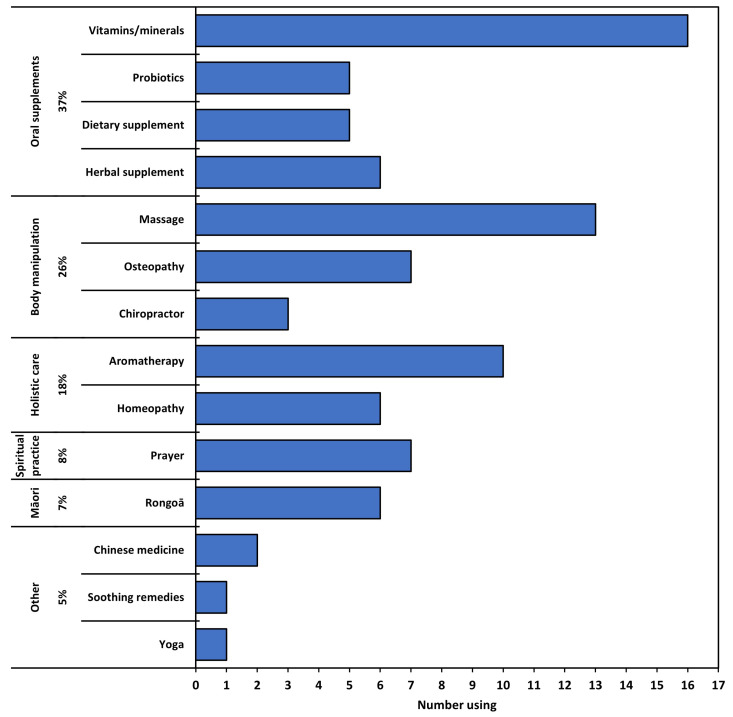
Types of CAM used by study cohort, grouped by type.

Of the 130 parents completing the survey, 59 (45%) reported that they use CAM therapies with their child attending hospital, with 18 (31% of users) using more than one type of CAM, giving a total of 88 CAMs used (Figure 1). Oral supplements and body manipulation methods were used most frequently, and 7% of parents used Rongoā Māori CAM, a traditional Māori healing system using plant-based remedies, massage, and spiritual healing. Twelve (57%) participants identifying as Māori used CAM, of which four (33%) used Rongoā Māori. 

Parents who used CAM with their child were asked for specific details for each CAM, namely the reasons for using, whether their child experienced side effects, and the frequency and duration of CAM (Table 2). CAM was most often taken daily by 37 (43%) and had been utilized for a duration of over 12 months by 46 (53%). The main reasons for using CAM for their child were for the treatment or prevention of symptoms, with only 25 (30%) using CAM for a chronic health condition. The majority of children (80 (92%)) experienced no side effects, and of those that did, all were classed as ‘mild’ (7 (8%)). Four experienced mild side effects from oral supplements, two from body manipulation, and one from ‘other’ CAM. The majority of CAM users saw improvement rated as ‘lots’ (36%) or ‘slight’ (52%).

### 3.3. Factors Associated with CAM Use by Children

#### 3.3.1. Socio-Demographic Variables

Only a few socio-demographic and health variables were associated with CAM use (Table 3). Parental and sibling use of CAM was associated with child CAM use. Children having needed emergency department (ED) care in the previous 12 months was associated with child CAM use. Further analysis showed no association between ED visits and the type of CAM used (all *p* > 0.05). 

#### 3.3.2. Socio-Economic Deprivation Deciles

**Figure 2:**
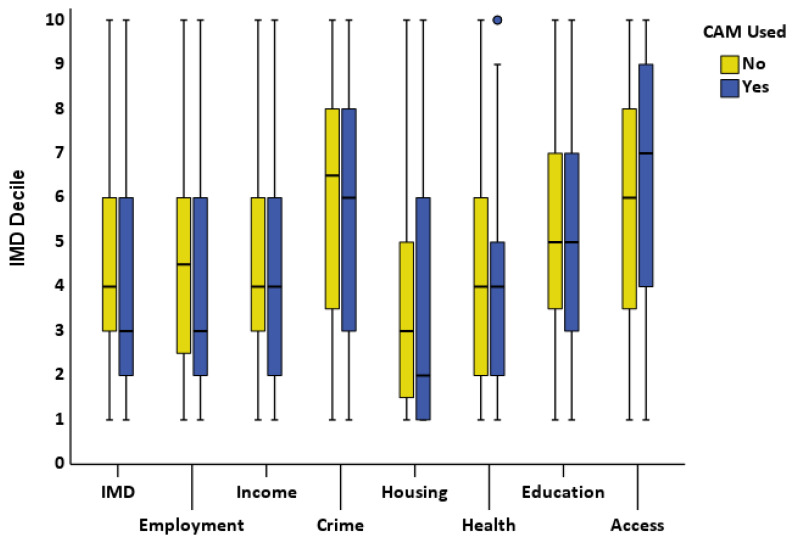
Difference between Index of Multiple Deprivation deciles for CAM users and non-CAM users. IMD = Index of Multiple Deprivation; CAM = Complementary and Alternative Medicine.

**Figure 3:**
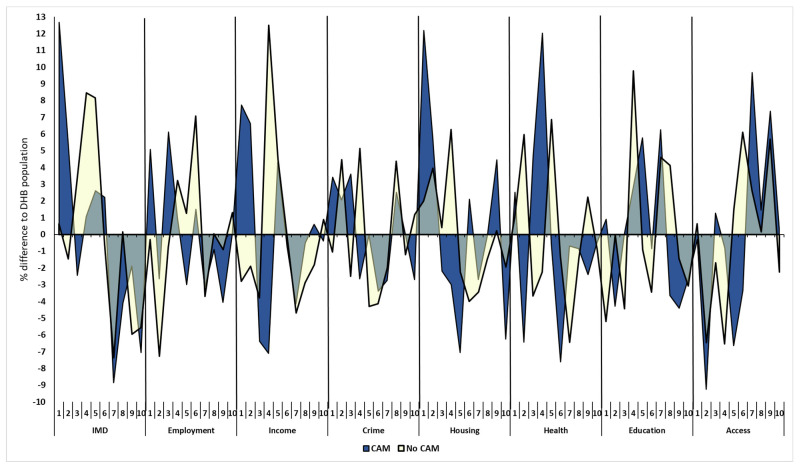
Comparison of deprivation deciles for CAM and non-CAM users with District Health Board data by residential address. DHB = District Health Board; IMD = index of multiple deprivation; CAM = Complementary and Alternative Medicine. Blue area represents CAM users, yellow non-users. Decile numbers represent the range of deprivation from low to high (1 = least, 10 highest).

No difference was seen in any of the Index of Multiple Deprivation (IMD) deciles between CAM users and non-CAM users (Figure 2). The IMD categories were then compared for CAM users and non-CAM users with normative data from the District Health Board (DHB) where each child resided (Figure 3). The percentage of CAM users in deprivation deciles differed from DHB data for overall IMD, income, housing, health, and geographic access (*p* < 0.001, 0.02, 0.03, 0.01, 0.001, respectively) but not for deciles of employment, crime, or education (*p* > 0.05 for all). The percentage of non-CAM users in deprivation deciles differed from DHB data for overall IMD, income, education, and geographic access (*p* < 0.001, 0.008, 0.02, 0.04, respectively) but not for deciles of employment, crime housing, or health (*p* > 0.1 for all).

#### 3.3.3. Parental Opinion

Parental opinion statements relating to efficacy, safety, and side effects were scored as ‘neutral’ by the majority, and most parents agreed that CAM therapists should be qualified and registered (Table 4). Many parents agreed that doctors should be supportive of CAM use. Parental opinion of the statement that they would only use CAM if a doctor recommended it influenced CAM use (*p* = 0.010). 

#### 3.3.4. Parental Health Literacy

For the cohort overall, the mean NVS score was 4.1 (SD 1.5) out of a maximum score of 6. NVS scores were not associated with parental education level (*p* = 0.21, CI 0.0 to 0.1), but were associated with household income (*p* < 0.001, CI 0.04 to 0.26), with the difference seen specifically between the lowest annual income bracket (up to 50,000 NZD) and brackets above 100,000 NZD. Parents identifying as NZ European had higher NVS scores than those not (mean difference (MD) −1.3, *p* < 0.001, CI −1.9 to −0.7), and those identifying as Asian had lower NVS scores than those not (MD 1.0, *p* = 0.02, CI 0.16 to 1.7), but no association was seen for other ethnicity groups (all *p* > 0.1). Parents who had higher health literacy were more likely to use CAM with their child than parents who did not use CAM (mean difference −0.58, *p* = 0.03). 

#### 3.3.5. Child Well-Being

**Figure 4:**
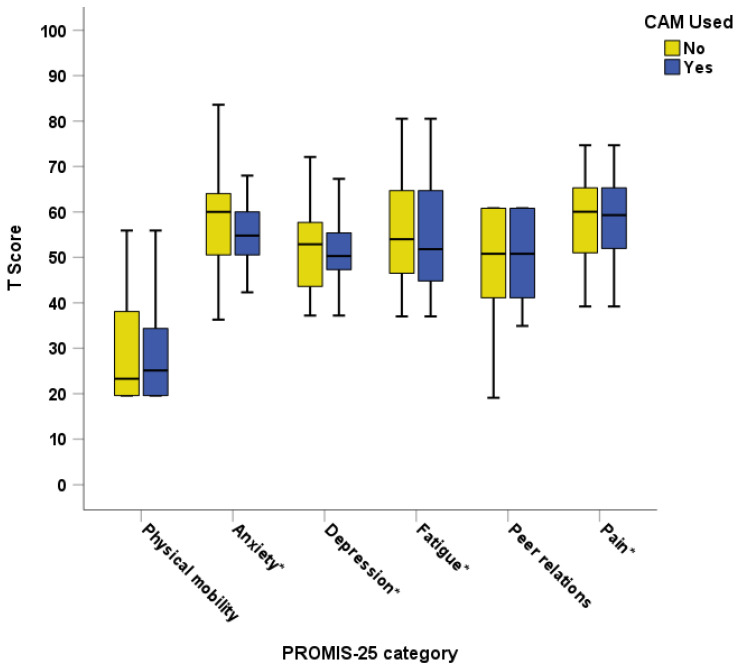
PROMIS-25 parent proxy measure domains compared for CAM users and non-CAM users. CAM = Complementary and Alternative Medicine; PROMIS = Patient-Reported Outcomes Measurement Information System; * indicates negative scale with higher scores meaning worse outcomes.

Seventy-eight (60%) children were over the age of five years and had the PROMIS-25 parent proxy assessment completed. No association was seen between CAM use and the different domains of the PROMIS-25 (Figure 4). There was also no association between pain intensity and the use of CAM (mean difference 0.31, *p* = 0.62). 

When analysing individual CAM types with the domains of the PROMIS questionnaire, there was an association between children using oral supplements and having a lower anxiety T-score (less anxiety) (mean difference 6.3, *p* = 0.02), but for no other PROMIS-25 domains. There was an association between the use of body manipulation and having higher mobility T-scores (better mobility) (mean difference −11.1, *p* = 0.006), but no other domains. No associations with PROMIS-25 scores were seen for the use of holistic, spiritual, Rongoā Māori, or other CAM. No association was seen between PROMIS-25 domain scores and the frequency or duration of CAM (all *p* > 0.05). 

### 3.4. CAM Disclosure Rates

Parents were asked if they disclosed their children’s CAM use to their child’s medical team, with the assumption that routine clinical assessment was carried out according to protocol in the clinical area they were recruited from. Only 35 (40% of all CAM users) had disclosed, representing a non-disclosure rate of 60%. The reasons given for non-disclosure were that they were not asked (25%), they did not think it was relevant/necessary/important (21%), it had not occurred to them (15%), they had not used it recently (11%), it was only vitamins/probiotics (8%), it was not being used for the reason they were in hospital (8%), and personal choice (4%), with 9% giving no reason for non-disclosure. Individual analysis CAM types showed that none of the CAM types had a significant association with disclosure (all *p* > 0.05). Those who did not disclose CAM use were found to have lower health literacy (NVS mean difference 1.07, *p* = 0.001). There was no association between disclosing parents and parental education (χ^2^ 1.6 (Phi 0.17), *p* = 0.65) or ethnicity (all *p* > 0.05).

## 4. Discussion

This study presents the prevalence of CAM use among a cohort of children attending a tertiary care hospital in New Zealand. Associations were seen between child CAM use and family CAM use, higher parental health literacy, and children attending the ED in the last 12 months. Over half of parents did not disclose their child’s CAM use to the medical team. 

The point prevalence of CAM in the current study (43%) is lower than that reported in a previous study among children in New Zealand (70%) [23] and recent research conducted at the same site as the current study (56%) [10]. The previous study at the same study site was conducted shortly after the New Zealand nationwide COVID-19 lockdown in 2020, and studies have suggested that COVID-19 resulted in an initial surge of CAM use due to the early lack of COVID-19 prevention and treatment options [40,41]. The development of vaccinations and public confidence in the current treatment for COVID-19 may have resulted in a subsequent decrease in CAM use, thus providing a possible explanation for the lower numbers of people using CAM in the current study. However, investigation of temporal trends and their influencing factors requires longitudinal data collection with repeated measures, rendering this a subjective observation. 

While the association between family use of CAM and child CAM use is a common finding throughout the literature [10,42], associations with healthcare visits, parental health literacy, and ethnicity have been infrequently addressed. In the current study, a relationship was shown between CAM use and higher parental health literacy. This finding supports the literature regarding health literacy and CAM use in adults: that those with higher health literacy levels are three times more likely to use relaxation techniques than those with lower levels [43], and overall more likely to use CAM [44]. However, there are limited studies exploring the association between parental health literacy and the use of CAM for their children. One study reported that the use of honey as CAM for children aged under one year has been associated with low parental health literacy [20]. Previous research has shown that there is an association between low parental health literacy and poor health outcomes for children with chronic illnesses [45], and between low health literacy and parental medication errors [46,47]. Parents need health literacy competencies to assess reliable health information in order to make informed decisions about their child’s health and well-being [48,49]. As it is known that health literacy can influence CAM use in adults, more research needs to be conducted to explore the association between parental health literacy and child CAM use in order to provide targeted education to reduce risk. In contrast to previous research in the same centre [10], the current study did not find that children of Māori ethnicity were more likely to use CAM, nor specifically Rongoā Māori. While this finding was not evident, assessing all outcomes to identify inequities for Māori participants in research studies remains a high priority in New Zealand within the clinical and research healthcare settings [50,51,52]. 

The current study found no association between CAM use and child well-being overall, as measured by the PROMIS-25 parent proxy tool. However, the association between specific CAM and PROMIS-25 domains is noteworthy. A previous study utilizing PROMIS-25 reported improved overall quality of life after children underwent chiropractic therapy with improvements in the domain of physical mobility [53]. This supports the findings in the current study, although the number of children using chiropractic treatment as a form of body manipulation was low at only 5% of those using CAM overall, and the numbers were not sufficient to allow for detailed analysis within the body manipulation sub-group by type. A previous systematic review highlighted that there is low-grade evidence of efficacy in the field of body manipulation for all paediatric age groups, and safety concerns have been reported, highlighting the importance of further research being conducted among children undergoing such treatments [54]. 

The association between oral supplements and lower anxiety levels in the current study also provides a direction for further research. While the prevalence of anxiety disorders among children worldwide is 6.5% [55], little research has been carried out to provide robust evidence of the efficacy of CAM for anxiety. While particular supplements are considered effective for specific patients [56], the lack of evidence means that CAM therapies are infrequently recommended in anxiety treatment guidelines [57]. While specific CAMs, such as the ayurvedic supplement ashwagandha, have been reported to be effective for anxiety among adults, albeit with low-grade evidence, trials among children have not been conducted [58]. With the variation in supplements reported in the current study, conclusions cannot be drawn on any possible associations between specific CAMs and anxiety, although one possibility may be that taking oral supplements induces a placebo effect. Studies on the placebo effect for children with anxiety disorders have shown response rates ranging from 40 to 50%, although this was generally reported as an early response that was not sustained [59].

The finding in the current study that children who presented to the ED in the last 12 months were more likely to use CAM is reflected in the wider literature that reports that CAM use is common among children attending EDs, at rates between 15% and 78% [8,60,61,62,63], similar to other healthcare settings. It is important to distinguish between presentations for emergency care due to CAM use and ED attendance while concurrently using CAM. Acute intakes of CAM requiring ED attendance among children have been shown to account for 8% of accidental medication ingestions (herbal and homeopathic remedies), and while the majority of children have few or no side effects from these, a proportion of CAM intoxications do require medical follow-up [64]. While the current study did not collect data on the reason for previous ED attendance, CAM use has previously been linked to poor outcomes for children with chronic conditions even though the direction of association is not always clear. In one study among children with asthma, CAM use was associated with an increased need for steroid therapy [65], and worse asthma control was associated with lower adherence to prescription medication [66]. The use of CAM by children with juvenile idiopathic arthritis has been associated with lower global health and physical functioning, although not with reduced adherence [67]. Children with atopic dermatitis using CAM have been reported as having a greater extent, severity, disease activity, and antibody levels than non-users [68]. Children with epilepsy using CAM have been reported to have increased seizure severity [69], and children with inflammatory bowel disease using CAM have been reported to have more severe disease activity [70,71]. It is unclear whether CAM use is associated with poor outcomes, or whether a child having more severe symptoms of a chronic condition may make them more likely to use CAM. Regardless, this further emphasizes the importance of healthcare practitioners asking parents of all children whether they use CAM, and of providing guidance on what constitutes CAM therapies and information regarding decision making and safety issues [72]. In the current study, many of the reasons parents gave for non-disclosure could indicate a lack of awareness of what constitutes CAM and also poor awareness of the safety implications of CAM use. 

### 4.1. Strengths

Children of indigenous Māori ethnicity were well represented in this study, therefore allowing cultural comparisons to be made regarding CAM use as in previous work [10]. The recruitment process took place in multiple clinical areas that allowed for analysis of the associations between CAM use and children with chronic illnesses or those taking prescription medications. 

### 4.2. Limitations 

This study had a smaller sample size than the previous study conducted in the same population, although recruitment exceeded the calculated required sample size. The assessment tool used for parental opinions was not validated. However, no appropriate validated tools were found. Utilizing the PROMIS-25 parent proxy tool excluded 40% of the study cohort from the child well-being analysis due to the age parameters of the assessment tool. This study was conducted at a single tertiary care hospital in New Zealand, which may affect the generalizability of the results to the wider paediatric population in New Zealand and beyond. A response rate was not able to be calculated that could enable comparisons with previous research. 

## 5. Conclusions

The current study provided important information on the use of CAM among this group of children in New Zealand and highlighted associations with parental health literacy that should be further explored. Parental opinions as well as poor disclosure rates have highlighted concerns regarding the safety of CAM. While these findings correspond with other literature, these should be addressed through improved awareness and education for parents to promote CAM disclosure, and for healthcare workers to encourage the inclusion of questions regarding all types of CAM in paediatric clinical assessments. Additional work to assess healthcare professionals’ opinions of CAM use for children, and the number asking parents about CAM use for their child, would add valuable insight. The current study adds to the growing evidence base of CAM use in New Zealand that may add strength to the call for legislation.

## Figures and Tables

**Table 1:** Patient demographic and health details for the overall cohort.

Variable	Category	Mean (SD) or N (%)
Child age	Years	6.7 (4.9)
Child sex	Male Female	66 (51) 64 (49)
Family ethnicity *	NZ European Māori Pacific MELAA Asian	105 (81) 21 (16) 4 (3) 5 (4) 15 (12)
Number of adults in family home	1 2 3 4–5	15 (12) 99 (76) 8 (6) 8 (6)
Number of children in family home	1 2 3 4–6	37 (29) 54 (42) 29 (22) 10 (7)
Child chronic health condition	No Yes Under investigation	87 (67) 29 (22) 14 (11)
Child on prescription medications	No Yes	83 (64) 47 (36)
Reason for attending hospital	Accident/injury New illness/condition Pre-existing condition Investigations	26 (20) 58 (45) 21 (16) 25 (19)
Parent age	Years	38.0 (7.7)
Parent sex	Male Female	19 (14) 111 (86)
Parent education level	High School College/vocational training University Post-graduate	41 (31.5) 20 (15.4) 46 (35.4) 23 (17.7)
Household income (NZD)	Up to 50,000 50,000–100,000 100,000–150,000 150,000–200,000 200,000+ Not stated	13 (10) 36 (28) 29 (22) 24 (19) 17 (13) 11 (8)
CAM used by parents CAM used by siblings	Yes Yes	81 (62.3) 62 (47.7)
Child health in last week	1–10 scale	6.3 (2.7)
ED visits in last 12 months PCP visits in last 12 months OPA visits in last 12 months	Yes Yes Yes	84 (64.6) 109 (83.8) 68 (52.3)

NZ = New Zealand; MELAA = Middle Eastern, Latin American, African; N = number; SD = standard deviation; CAM = Complementary and Alternative Medicine; NZD = New Zealand dollars; ED = emergency department; PCP = primary care physician; OPA = outpatient appointment. * participants could choose more than one answer.

**Table 2:** Reasons for CAM use among 59 children using 88 CAMs, alongside perceived benefit, side effects, duration, and frequency of CAM.

Variable	Category	Frequency N (%)
Used for a chronic condition	Yes No	25 (30) 58 (70)
Side effects	None	80 (92)
	Mild	7 (8)
	Moderate	0
	Severe	0
Benefits seen	None	10 (12)
	Improved slightly	44 (52)
	Improved lots	31 (36)
Reason for using *	Treatment of symptoms	56 (39)
	Prevention of symptoms	37 (26)
	To complement conventional treatment	21 (14)
	Knowledge of it working for other people	15 (10)
	Lack of conventional treatment	5 (3)
	Worry about side effects from conventional treatment	4 (3)
	Lack of confidence in conventional treatment	4 (3)
	More effective than conventional treatment	3 (2)
Duration	More than 12 months	46 (53)
	6–12 months	12 (14)
	1–6 months	19 (22)
	Less than 1 month	10 (11)
Frequency	When needed	21 (24)
	Yearly	0
	Every 6 months	8 (9)
	Monthly	1 (1)
	Weekly	20 (23)
	Daily	37 (43)

* more than one option could be chosen.

**Table 3:** Associations between socio-demographic and health variables with CAM use.

Variable	Mean Difference or χ^2^ (Phi)	*p*-Value	95% CI
Child age	−1.0	0.25	−2.7, 0.7
Parent age	−1.3	0.33	−4.0, 1.4
Overall health rating	0.0	0.99	−0.9, 0.9
Child gender	0.1 (−0.0)	0.72	−0.1, 0.2
Ethnicity NZ European	1.1 (0.1)	0.30	−0.2, 0.1
Ethnicity Māori	1.4 (0.1)	0.24	−0.2, 0.1
Ethnicity Pacific	1.5 (0.1)	0.23	−0.1, 0.0
Ethnicity MELAA	0.4 (0.1)	0.51	−0.1, 0.0
Ethnicity Asian	2.4 (−0.1)	0.12	−0.0, 0.2
Number of adults in family home	1.2 (0.1)	0.76	0.8, 0.8
Number of children in family home	0.2 (0.0)	0.97	0.9, 0.9
Child chronic health condition	0.9 (0.1)	0.42	−0.3, 0.1
Child on prescription medications	1.0 (0.1)	0.33	−0.3, 0.1
Reason for attending hospital	1.9 (0.1)	0.72	−0.5, 0.3
Parent gender	3.3 (0.2)	0.07	−0.2, 0.1
Parent education level	7.3 (0.2)	0.77	−0.4, 0.3
Household income (NZD)	0.9 (0.1)	0.75	−0.4, 0.5
CAM used by parents	26.8 (0.5)	<0.001	−1.3, −0.7
CAM used by siblings	59.4 (0.7)	<0.001	−1.6, −0.9
ED visits in last 12 months	4.7 (0.2)	0.03	−0.3, 0.0
PCP visits in last 12 months	0.1 (−0.0)	0.82	−0.1, 0.1
OPA visits in last 12 months	0.1 (−0.0)	0.76	−0.1, 0.2

CI = confidence interval; NZ = New Zealand; MELAA = Middle Eastern, Latin American, African; N = number; CAM = Complementary and Alternative Medicine; ED = emergency department; PCP = primary care physician; OPA = outpatient appointment.

**Table 4:** Results of opinion survey [10] and associations between statements and CAM use by children.

Opinion Statement	CAM Use	Disagree N (%)	Neutral N (%)	Agree N (%)	χ^2^ (Phi)	*p* Value
Doctors should be supportive of people using CAM	No CAM CAM	0 (0) 0 (0)	18 (24) 7 (14)	55 (76) 50 (86)	2.2 (0.1)	0.14
Doctors should ask patients if they are using CAM	No CAM CAM	0 (0) 0 (0)	16 (23) 11 (19)	57 (77) 46 (81)	0.3 (0.0)	0.59
Doctors should know about CAM and be able to give advice	No CAM CAM	2 (3) 1 (2)	15 (21) 12 (20)	56 (76) 44 (78)	0.2 (0.0)	0.91
I would only use CAM for my child if a doctor recommended it	No CAM CAM	24 (34) 33 (56)	20 (27) 15 (27)	29 (39) 9 (17)	9.2 (0.3)	0.01
CAMs do not interfere with prescribed drugs	No CAM CAM	13 (17) 15 (27)	41 (56) 24 (42)	19 (27) 18 (31)	0.2 (0.2)	0.23
Enough is known about the effectiveness of CAM	No CAM CAM	26 (34) 21 (39)	34 (48) 19 (32)	13 (18) 17 (29)	3.7 (0.2)	0.16
Enough is known about the safety of CAM	No CAM CAM	15 (20) 14 (26)	37 (51) 18 (32)	21 (29) 25 (42)	5.6 (0.2)	0.10
Enough is known about the side effects of CAM	No CAM CAM	17 (21) 14 (27)	38 (54) 19 (32)	18 (25) 24 (41)	6.2 (0.2)	0.05
There is sufficient information available about CAM	No CAM CAM	28 (38) 22 (39)	29 (39) 16 (29)	16 (23) 19 (32)	2.2 (0.1)	0.34
CAMs have fewer side effects than prescribed or conventional treatment.	No CAM CAM	4 (6) 6 (10)	47 (63) 24 (44)	22 (31) 27 (46)	4.9 (0.2)	0.09
CAM is more effective than prescribed or conventional treatment	No CAM CAM	26 (37) 12 (20)	42 (56) 38 (68)	5 (7) 7 (12)	4.4 (0.2)	0.11
CAM therapists/practitioners should be qualified and registered.	No CAM CAM	1 (2) 1 (2)	13 (18) 13 (22)	59 (80) 43 (76)	0.3 (0.0)	0.86
CAM is used by people due to a lack of conventional treatment for an illness or condition.	No CAM CAM	38 (52) 31 (54)	27 (38) 18 (31)	8 (10) 8 (15)	1.3 (0.1)	0.52
CAM is used by people due to a lack of conventional treatment for an illness or condition	No CAM CAM	26 (35) 22 (39)	33 (47) 23 (39)	14 (18) 12 (22)	0.8 (0.1)	0.68
CAM can be used to replace conventional treatment	No CAM CAM	26 (35) 24 (43)	32 (44) 21 (37)	15 (21) 12 (20)	0.8 (0.1)	0.68
The cost of CAM puts people off using it	No CAM CAM	11 (15) 11 (18)	24 (34) 24 (41)	38 (51) 22 (41)	1.3 (0.1)	0.52

## Data Availability

Data are available upon reasonable request to the corresponding author A.V.R.
